# Responding to Other People’s Posture: Visually Induced Motion Sickness From Naturally Generated Optic Flow

**DOI:** 10.3389/fpsyg.2018.01901

**Published:** 2018-10-08

**Authors:** Henry E. Cook, Justin A. Hassebrock, L. James Smart

**Affiliations:** Department of Psychology, Miami University, Oxford, OH, United States

**Keywords:** motion sickness, posture, optic flow, perception and action, virtual reality, head mounted displays

## Abstract

Understanding the relationship between our actions and the perceptual information that is used to support them is becoming increasingly necessary as we utilize more digital and virtual technologies in our lives. Smart et al. (2014) found that altering the relationship between perception and action can have adverse effects, particularly if the perceptual information cannot be used to guide behavior. They also found that motion characteristics varied between people who remained well and those that became motion sick. The purpose of this study was to determine the influence of naturally produced virtual motion on postural regulation and examine how people respond to different types of optical flow (produced by other people). Participants were either exposed to optic flow produced by the postural motion of a person who did not become motion sick, or a person who did exhibit motion sickness from Smart et al. (2014). It was discovered that participants exhibited both stronger coupling and more incidents of motion sickness in response to optic flow generated by a non-sick participant. This suggests that participants recognized the potentially usable nature of the well-produced optic flow- but the open loop nature of the stimuli made this perception disruptive rather than facilitative.

## Introduction

IMAX (large screen format) theaters, high definition television, immersive virtual and gaming environments, as well as commercial grade head-mounted displays (HMDs) are becoming increasingly commonplace technologies that are expanding the realm of possibilities for novel experiences and interactions, while at the same time facing some enduring challenges for widespread successful engagement. One of the most common challenges is the potential for motion sickness and similarly documented ailments (e.g., cyber sickness, simulator sickness); particularly when depicting some form of self-motion. Further complicating this issue is that “simply” improving the technology does not mitigate this issue and may in many instances make it more prevalent (Biocca, 1992; Palmisano et al., 2017). Thus solutions to preventing motion sickness may reside within changing how virtual technology is implemented rather than how it is designed. Successfully changing implementation necessitates understanding the relationship between our actions and the perceptual information that is used to support them.

Exploring the link between perception and action in the context of motion sickness can be traced back to Riccio and Stoffregen (1991) *Postural Instability* theory, which states that poor outcomes such as motion sickness should be characterized as perception-action problems rather than perceptual-processing issues (such as sensory conflict theory; Reason and Brand, 1975; Oman, 1990*)*. Simply put, Riccio and Stoffregen (1991) assert that motion sickness and other negative outcomes emerge from degraded postural control strategies (i.e., instability) that develop over time, rather than a cognitive inability to resolve sensory conflicts. Since proposing postural instability as a causal mechanism, researchers have provided support by demonstrating that the manipulation of visual stimulation (i.e., optic flow) can perturb postural stability and in turn produce an increase in subsequent reports of motion sickness (Stoffregen and Smart, 1998; Stoffregen et al., 2000; Smart et al., 2002, 2007; Villard et al., 2008). These researchers were able to produce disruptions in participants’ actual postural motion by exposing participants to computer-generated motion; in these studies, a sum of 10 sine waves that simulated the optic flow that is typically produced by postural sway. Importantly, these studies were able to show that postural disruptions occurred prior to reports of motion sickness symptomology.

In an extension of this paradigm, Smart et al. (2014) sought to determine how changes in the complexity of optic flow and changes in the manner of behavioral coupling (i.e., the relation between available optical information and participants’ physical motion) influence differences in postural regulation between *Motion Sick* individuals and *Well* participants. The researchers manipulated the complexity of optic flow by presenting simple sinusoidal motion or naturally generated (from the participants’ own movements), complex motion. Coupling was manipulated by either playing back previously recorded sway, or generating flow in real time based on the participants’ movements. In the real time conditions, the relationship between sway and optic flow was either anti-phase (what we normally experience) or in-phase (where moving forward produces contraction rather than expansion of the stimuli). It was found that incidences of motion sickness increased with more complex motion and when the behavioral coupling was altered (in-phase) or when the researchers presented participants with their own motion, decoupled (i.e., not real-time). Interestingly what this study revealed were differing patterns of postural motion/structure for *Well* participants and *Motion Sick* participants. What was discovered is that postural motion preceding reports of motion sickness tends to increase in magnitude and spatial complexity over time while remaining temporally rigid (motion pattern persists over time, once the disruption occurs the participants do not recover), while postural motion for those that remain well tend to exhibit the opposite trend. Importantly in this study, each participant’s motion was reflected in the structure of optic flow presented in the virtual environment (VE). This raises the question of whether these patterns of optic flow are inherently facilitative or disruptive, that is, do they carry the “information” for successful (or unsuccessful) behavioral regulation?

The current study was designed to examine the behavioral characteristics exhibited by participants who are exposed to other people’s postural (OPP) motion. In particular, our goal was to determine how participants respond to OPP; specifically can they utilize OPP to successfully regulate their own postural sway? We addressed these questions by exposing participants to two types of optical motion in a VE by way of a HMD. Participants were exposed to optical flow created from (1) postural motion previously recorded from an individual in a previous study (Smart et al., 2014) who successfully completed her/his postural regulation trials without reporting motion sickness (Well-flow) or, (2) postural motion previously recorded from an individual who completed his/her postural regulation trials but reported motion sickness (Sick-flow). The specific trials employed in this study were chosen because their motion parameters (PL, EA, PL_N_, SEn) closely matched the overall means obtained by Smart et al. (2014) for *Well* and *Motion Sick* participants.

While we expected based on the results of Smart et al. (2014) that both conditions should produce motion sickness, it was not theoretically clear which condition (Well-flow or Sick-flow) should have produced higher incidences of motion sickness. Following the wave interference hypothesis (Stoffregen and Smart, 1998; Smart et al., 2002) that suggests that the interaction of similar waveforms will result in greater instability, we expected that the Well-flow condition would produce higher rates of motion sickness because its structure would likely be close to that that could be produced by the current participants (prior to incidences of instability/sickness, which develop over time). However, Smart et al. (2014) found that the greatest incidence of motion sickness occurred in the condition that had the least informative stimuli suggesting that the Sick-flow condition would be likely to produce higher rates of sickness given the likelihood that the information (structure) provided in this stimuli would not be supportive of successful postural regulation. Fortunately, this is an empirical question that can be addressed by the current study.

Whatever the incidence rate between conditions, we expected to find a similar divergence in the postural sway dynamics between motion sick participants and those who remain well as was found by Smart et al. (2014). The same set of postural sway measures employed by Smart et al. (2014) [Path Length (PL), Elliptical Area (EA), Normalized Path Length (PL_N_), and Sample Entropy (SEn)] were utilized in the current study.

Finally, to address the utility of the “information” provided by OPP we assessed the degree to which participants coupled or become entrained with the stimuli. We hypothesized that Well-flow, which represents a person who successfully regulated his or her sway, would potentially allow for easier regulation of sway in the VE. In contrast, Sick-flow, which depicts motion from a person who became unstable (and subsequently motion sick), would provide insufficient information for regulation. Thus we expected to see less coupling in the Sick-flow condition.

To determine whether coupling differs between optic flow conditions, a set of non-linear synchronicity analyses were conducted on the postural sway data. The analyses used to determine synchronized behavior include; (1) Average Mutual Information (AMI), which examines the amount of information shared (dependency) between two time series (Thomas et al., 2014); (2) Cross-Correlation (CC), which determines how linearly correlated two time series are while accounting for time lags between stimuli and response (Strang et al., 2014); (3) Coherence (CoH), which examines frequency coupling (similarity) across the two time series; and (4) Cross-Fuzzy Entropy (CFEn), which determines temporal stability of the coupling between two time series (Strang et al., 2014). We expected that higher AMI, CC, and CoH as well as lower CFEn values would indicate stronger coupling with the optic flow. Given this we predicted that coupling should be higher in the Well-flow condition and with participants who remain well.

## Materials and Methods

### Participants

Forty participants (19 male, 20 female, and one participant who did not specify gender) drawn from the psychology department participant pool were randomly assigned to one of two conditions: Well-flow (10 male, 10 female), and Sick-flow (9 male, 10 female, 1 undisclosed). None of the current participants were involved in the studies reported in Smart et al. (2014). Male participants had a mean (SE) height of 1.81 (0.02) m and weight of 78.23 (2.67) kg, while female participants had a mean (SE) height of 1.69 (0.01) m and weight of 62.43 (1.56) kg. Participants reported being in their normal state of health, and had normal or corrected to normal vision. No participants reported any history of falls, dizziness, or vestibular dysfunction and all participants were able to stand on 1 ft for 30 s with their eyes closed. Participants were instructed not to eat 2 h prior to their experimental session and compliance with this request was verified at the beginning of the sessions. Participants received course credit for their time and were aware that they could cease participation at any time and for any (or no) reason without loss of benefits. As part of the informed consent process, participants were made aware that the experiment could have produced mild motion sickness, but were unaware of the specific hypotheses of the study. The study protocols were approved by the Miami University Institutional Review Board (#00116r). All participants gave written consent in accordance with the Declaration of Helsinki.

### Materials

Materials used in this study were the same as employed by Smart et al. (2014) and described below. The single deviation from the original study involves the baseline stimulus which is discussed in the procedure.

#### Questionnaires

Two different questionnaires were used in this study. The first asked for basic demographic information, motion sickness history and perceived susceptibility to motion sickness (10 point scale with one being not susceptible and 10 being very susceptible). The second questionnaire was the widely used and accepted simulator sickness questionnaire (SSQ; Kennedy et al., 1993), which determines the level of common motion sickness symptoms prior to exposure and the extent to which immersion in a VE subsequently produces and/or elevates those symptoms (determination of sick/well was by verbal report of the participants, not their score on the SSQ).

#### Postural Sway Measurement

A magnetic tracking system was used to record the postural sway of participants (Flock of Birds; Ascension, Inc., Burlington, VT, United States) in the anterior-posterior (AP) and medial-lateral (ML) planes. The system consisted of an emitter that created a low-level magnetic field extending 1 m in radius. A sensor was placed on the top of the participant’s head and held in place with athletic prewrap. The AP and ML motion of the sensor disturbed the magnetic field, and these disturbances were then recorded by the computer at a sampling rate of 50 Hz.

#### Head Mounted Display (HMD)

One pair of virtual i-glasses SVGA 3D ASO1317 (I-O display systems, CA) personal displays were used to present the VE. The displays simulated a 1.78 m screen (diagonally) that is 3.96 m away from the viewer’s eyes resulting in a field of view of 24° (diagonally). The HMDs were only partially immersive (participants could see the lab below and peripherally). Thus, during exposure, the laboratory lights were turned off.

#### Virtual Environment

**FIGURE 1:**
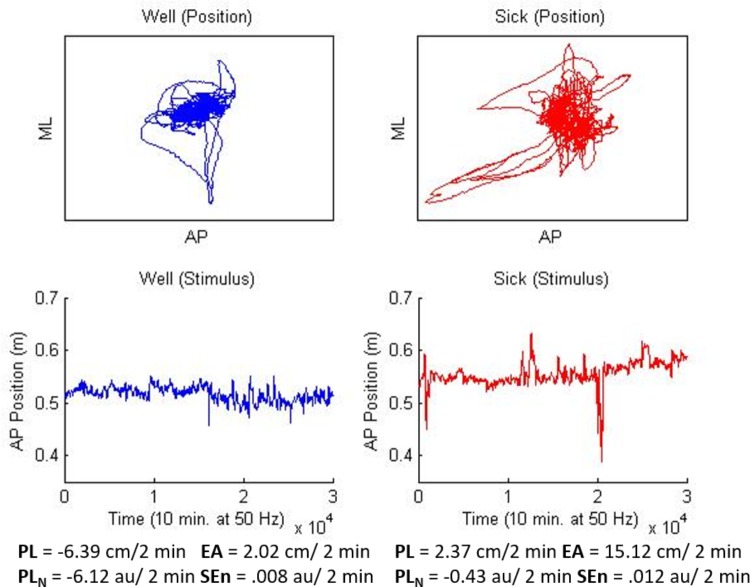
Stimuli used for Well-flow (blue/left) and Sick-flow (red/right) conditions. Top panels depict two dimensional motion (10 min) for each participant, lower panels depict, AP motion only, the waveform that was used to generate the current stimuli. Values at the bottom represent slope means for each stimulus and match the overall means from Smart et al. (2014).

The VE consisted of a spherical “star field” consisting of a pattern of randomly placed white dots on a black background in the shape of a sphere. The sphere was positioned such that participants were “standing” in the center of the sphere with “stars” located at a starting distance of about 3.3 m away. The stars in the field were made to translate in the AP plane for all conditions and trials. In addition, stars would change from white to red for a period of 3 s at quasi-random intervals during experimental trials (14 shifts in each trial) and were used in the manipulation check to ensure that participants were engaged in the task. Following Smart et al. (2014) AP motion of the star field was amplified (15x) relative to the motion of the participant so that visual change was both observable and smooth. The motion path for the star field was generated from the data of two participants’ last experimental trial from Smart et al. (2014); one who did not become motion sick, and one who did report motion sickness. The data was chosen because the sway properties of these two participants most closely matched the overall pattern of results discovered by Smart et al. (2014). The Well participant’s data showed decreases over time in PL, PL_N_, and EA coupled with relatively higher SEn. The Sick participant’s data exhibited the opposite pattern (the general finding of Smart et al., 2014; see **Figure 1**).

#### Hardware/Software

One computer (Dell Optiplex GX270) was used in this study to display the stimulus through the HMD and simultaneously record the postural sway of participants. Participants’ sway was recorded using the same software package that was used to create the star field stimuli and display it to participants (Vizard; version 2.53; World Viz, Santa Barbara, CA, United States).

### Procedure

Upon entering the lab, participants were presented with a consent form that explained the purpose of the experiment and their rights. After signing the consent form, participants were asked to complete the two questionnaires described above (i.e., SSQ and sickness history). Also, participants were asked to keep the symptoms described in the SSQ in mind during the experiment, and in the event of an increase or emergence of these symptoms, to inform the researchers immediately so that the experiment could be halted.

For safety, and to ensure that participants had comparable balance capabilities prior to exposure, participants were asked to complete two balance checks. The first involved walking a line in heel-toe fashion (standard field sobriety test) and the second had participants stand on their preferred leg with their eyes closed for 30 s. If a participant was unable to complete either of these checks, she or he was excused from the study. No participants were excluded from the study as a result of the balance checks.

The experiment consisted of up to three trials (depending on whether the participant became motion sick), each with a duration of 10 min. The first trial was used to assess baseline (with static computer-generated stimulus) postural sway, during which the participants stood bipedally in the lab. The stimulus was the star field sphere zoomed out in the HMD so that it appeared to be a flat circle of white dots in an otherwise black background about 0.5 m in diameter. The star field was static during this baseline trial, however, it did still shift occasionally from white to red.

Following the baseline trial, up to two experimental trials (20 min of exposure) were conducted, depending on whether the participants became motion sick via self-report. This a reduction in trials from Smart et al. (2014) as they noted that the majority of motion sickness reports occurred by the end of the second trial. In the Well-flow condition participants were exposed to optical flow generated from the postural motion of a participant who did not become motion sick in Smart et al. (2014) study. In the Sick-flow condition participants were exposed to optical flow generated from the postural motion of a participant who became motion sick in Smart et al. (2014) study. Both conditions were open-loop presentations in that the participants’ current movements did not impact the optical motion generated in the HMD. The well and motion sick data were chosen by the experimenters and reflected the general motion profiles for well and motion sick participants obtained by Smart et al. (2014) (see **Figure 1**). In the experimental trials, the participants were asked to remember how many times the stars in the field changed from white to red (color shift task; a manipulation employed to ensure that they were paying attention to the stimuli). At the conclusion of each trial, the participants were asked (1) how many times the stimuli changed from red to white and (2) how the participants felt (if they experienced any symptoms of motion sickness).

In the event that the participants indicated symptoms of motion sickness, the experiment was stopped (even if it was in the middle of a trial). The participants once again filled out the SSQ indicating the new level of their symptoms. They were allowed to rest and asked to stay in the laboratory for observation for 15 min. After this time, if the participants felt better, they were allowed to leave after successfully repeating the two balance checks. If the participants had no symptoms of motion sickness at any time during the trials, they were asked to complete the SSQ after completing the last trial. As before, the participants were only allowed to leave after successful completion of the balance checks. In either case, the participants were given a third copy of the SSQ. In the event that the participants exhibited symptoms at some time (up to 24 h) after leaving the laboratory, they were asked to fill out the questionnaire at that time and return it. If the participants had no symptoms, they were asked to complete and return the questionnaire approximately 24 h after completing the experiment.

## Results

As mentioned in the introduction the purpose of this analysis is threefold; (1) to determine if the optic flow generated by previous *Motion Sick* and *Well* participants produce different rates of motion sickness, (2) to determine if similar divergences in postural sway characteristics emerge between current *Motion Sick* and *Well* participants as found in Smart et al. (2014), and (3) to determine if the optic flow generated by previous *Motion Sick* and *Well* participants differentially influence the postural regulation of participants in the current study. To address these questions, we recorded motion sickness incidence rates and symptomology, and analyzed both structural and temporal properties of participants’ postural motion individually as well as in relation to the stimuli (i.e., coupling). As in Smart et al. (2014), postural motion was analyzed in 2 min windows (such that a person completing both experimental trials would have 10 values for each measure). As in the previous research a linear slope was derived from the trend line created by the measures at each time window (i.e., the value obtained from each 2 min window, five values per 10 min trial) as an additional index of how regulation evolved over time.

### Color Shift Performance (Manipulation Check)

For the baseline trial all participants regardless of condition correctly identified the number of color shifts (14). For the experimental trials, a 2 (Condition) × 2 (Health) between-groups ANOVA revealed a significant main effect for Health, *F*_(1,36)_ = 8.62, *p* < 0.05, ${\text{η}}_{\text{p}}^{2}$ = 0.19. *Well* participants [*M(SE)* = 14 (0.3), 100% accurate] were more accurate in detecting red shifts than *Motion Sick* participants [*M(SE)* = 12.34 (0.5), 88% accurate]. Flow condition did not significantly influence accuracy although participants in the Well-flow condition [*M(SE)* = 13.5 (0.4), 96% accurate] were slightly more accurate than those in the Sick-flow condition [*M(SE)* = 12.9 (0.4), 92% accurate].

### Motion Sickness History and Incidence

Participants were asked to rate their susceptibility to motion sickness on a 10 point scale (with 10 being very susceptible) prior to exposure to the stimuli. Participants who became motion sick in the current study reported a mean (SE) susceptibility of 3.08 (0.58) out of 10; participants who remained well reported a mean (SE) susceptibility of 3.64 (0.39) out of 10. This difference was not significant. In addition, reported susceptibility did not differ significantly between males [3.32 (0.48)] and females [3.55 (0.46)] nor between Well-flow [3.60 (0.51)] and Sick-Flow [3.35 (0.40)].

Overall there were 12 (6 male and 6 female) explicit reports of motion sickness (30%). Five (42%) of these participants reported past motion sickness. Notably, the majority of sickness reports [8 participants (67%)] occurred during exposure to the Well-flow stimulus (5 male and 3 female, 40%). In the Sick-flow condition 1 male and 3 females (20%) reported motion sickness. A chi-squared analysis of the incidence rates revealed that they were not significantly different from the average incidence rate of 42% for visually induced motion sickness studies (Playback and Normal Coupling conditions – Stoffregen and Smart, 1998; Smart et al., 2002, 2014; Villard et al., 2008), nor were they significantly different from each other.

### SSQ

**FIGURE 2:**
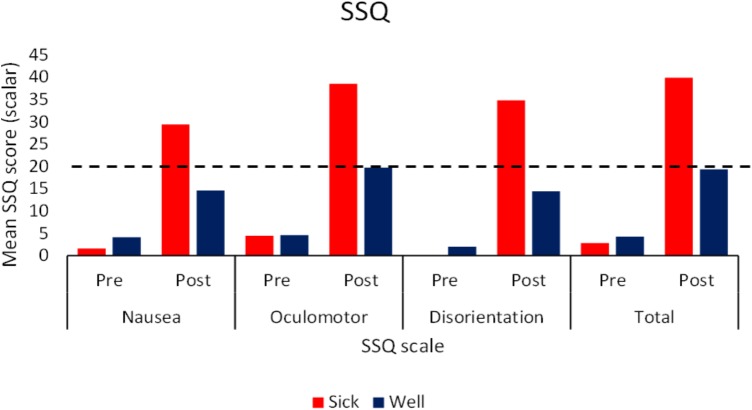
Mean pre and post exposure SSQ scores (subscale and total) as a function of participants’ health status. Dotted line indicates typical severity that indicates motion sickness (Kennedy et al., 2003; *N* = 40).

Simulator Sickness Questionnaire data for the two flow conditions were analyzed together. Pre-Post (Wilcoxon Signed Rank test), and Sick-Well (Mann–Whitney U test) comparisons were performed. We also ran a comparison analysis across flow conditions (Mann–Whitney U test) averaging over health of the participant. While Kennedy et al. (1993) developed a method for normalizing SSQ scores, since the original data is at best ordinal level measurement, we felt that non-parametric statistics were more appropriate to run in this case. The analyses revealed that pretest scores did not differ significantly between *Motion Sick* and *Well* participants, or between flow conditions, for any of the subscales or total SSQ scores. However, posttest scores for each subscale as well as the total score differed significantly for *Motion Sick* and *Well* participants (*p* < 0.05). The magnitude of reported symptom severity by the participants who self-identified as motion sick across conditions (See **Figure 2**) was comparable to those typically reported in VEs (typical range is 19–55; Kennedy et al., 2003). There was a significant difference (*p* < 0.05) in Oculomotor post subscales scores between those exposed to Well-flow (30.18) and those exposed to Sick-flow (19.71). There were no differences between *Motion Sick* participants in the two conditions nor were there differences between the *Well* participants in the two conditions. It should be noted that in general post test scores (both total and subscale) were significantly higher (*p* < 0.05) than pretest scores (regardless of health) suggesting that while only 30% of the participants explicitly reported motion sickness, nearly all participants reported increases in symptomology. This highlights the caution that should be taken with relying on SSQ responses as the main tool used to determine motion sickness.

### Postural Response

As in Smart et al. (2014), we examined two measures of magnitude (PL and EA) and two measures of structure (PL_N_ and SEn) to determine if there were characteristic sway differences between sick and well participants. As in Smart et al. (2014) we calculated these measure for each 10 min trial in 2 min windows (corresponding to 6000 data point time-series) such that if participants completed both experimental trials they would have 10 values for each measure. Replicating the analysis of Smart et al. (2014) we analyzed both raw values and derived slopes over the trials/windows to look for trends that may emerge over time. Three way mixed ANOVAs [Condition (2) × Health (2) × Window (5)] were performed on the raw data and Two way between ANOVAs [Condition (2) × Health (2)] were performed on the slope measures.

#### Baseline Trial (No Stimulus Movement)

Analysis of the raw values revealed significant effects of time window for all four postural response measures. PL *F*_(4,140)_ = 7.56, *p* < 0.05, ${\text{η}}_{\text{p}}^{2}$ = 0.18, and PL_N_ *F*_(4,140)_ = 12.76, *p* < 0.05, ${\text{η}}_{\text{p}}^{2}$ = 0.27 both exhibited *u*-shaped patterns with higher values during the first 2 min (0–2 min) window and last 2 min (8–10 min) window. **EA** *F*_(4,140)_ = 7.46, *p* < 0.05, ${\text{η}}_{\text{p}}^{2}$ = 0.07 and **SEn** *F*_(4,140)_ = 6.97, *p* < 0.05, ${\text{η}}_{\text{p}}^{2}$ = 0.17 both showed linear increases over time windows. There were no significant differences for Flow condition (Well-flow, Sick-flow), Participant Health (*Well*, *Motion Sick*), nor the interaction between Condition and Health.

#### Path Length (PL)

**FIGURE 3:**
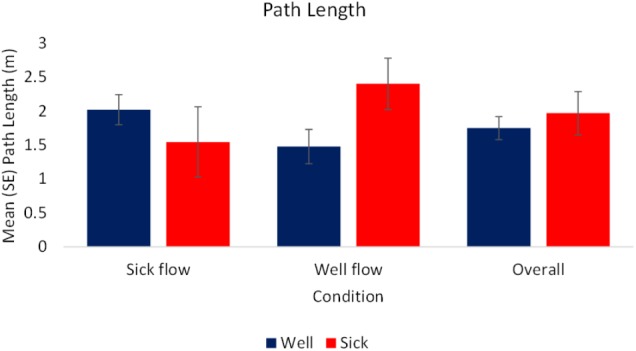
Mean (SE) Path Length as a function of Condition and Health (*N* = 40).

The analysis of the raw values revealed a significant interaction between condition and health, *F*_(1,70)_ = 3.74, *p* < 0.05, ${\text{η}}_{\text{p}}^{2}$ = 0.05. In the Well-flow condition *Motion Sick* participants [*M(SE)* = 2.4 (0.38) m] moved to a greater extent than the *Well* participants [*M(SE)* = 1.48 (0.25) m]. In the Sick-flow condition the opposite pattern emerged with *Well* participants [*M(SE)* = 2.02 (0.22) m] exhibiting more sway than *Motion Sick* participants [*M(SE)* = 1.55 (0.52) m]. See **Figure 3** for a depiction of these results. There were no other significant effects for the raw values, nor were there any significant effects revealed by the analysis of the slope data.

#### Elliptical Area (EA)

**FIGURE 4:**
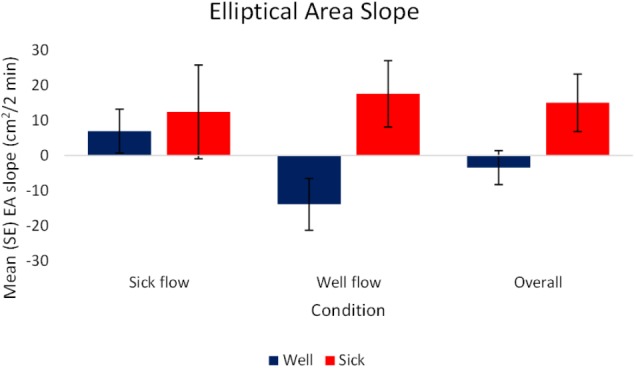
Mean (SE) Elliptical Area Slope as a function of Condition and Health (*N* = 40).

The analysis of the slope data revealed a significant effect of health, *F*_(1,72)_ = 3.77, *p* < 0.05, ${\text{η}}_{\text{p}}^{2}$ = 0.05. *Motion Sick* participants [*M(SE)* = 15.01 (8.18) cm^2^/2 min] exhibited a higher rate of change in magnitude of motion over time while *Well* participants [*M(SE)* = -3.45 (4.83) cm^2^/2 min] exhibited a lower rate of change in magnitude of motion over time. This indicates that *Motion Sick* participants generated larger movement overall and at relatively faster rate than *Well* participants. The *Well* participants tended to decrease their movement overall, doing so at a slower rate. See **Figure 4** for a depiction of these results. There were no other significant effects, nor were there any significant effects revealed by the analysis of the raw values.

#### Normalized Path Length (PL_N_)

The analysis of the raw values revealed a significant effect of window (time), *F*_(4,280)_ = 10.74, *p* < 0.05, ${\text{η}}_{\text{p}}^{2}$ = 0.13. This effect was produced by the complexity of motion being significantly lower in the second 2 m window [minutes 2–4; *M(SE)* = 81.28 (4.66) a.u.] than the other time windows which did not differ significantly [minutes 0–2 and 5–10; *M(SE)* = 92.56 (4.94) a.u.] regardless of health or condition. No other significant effects were revealed, nor were there significant effects revealed by the analysis of the slope data.

#### Sample Entropy (SEn)

The analysis of the raw values revealed a significant effect of window (time), *F*_(4,284)_ = 8.13, *p* < 0.05, ${\text{η}}_{\text{p}}^{2}$ = 0.1. SEn values increased over windows regardless of flow condition or health status of the participants. The increase occurred between the first 2 min window [minutes 0–2; *M (SE)* = 0.17 (0.01)] and third 2 min window [minutes 4–6; *M (SE)* = 0.2 (0.0)]. This indicates that participants’ movement strategies became more variable during the early stages of the trials. No other significant effects emerged, nor were there any significant findings from the analysis of the slope data.

### Postural Coupling

#### Average Mutual Information (AMI)

**FIGURE 5:**
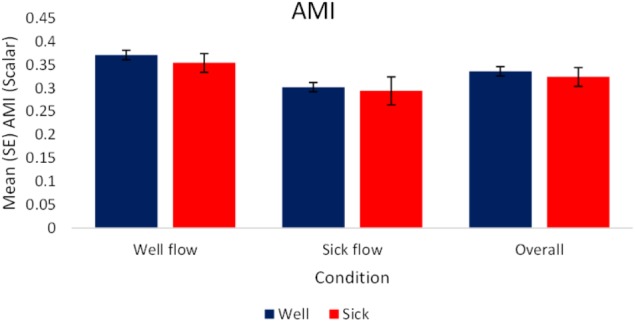
Mean (SE) Average Mutual Information as a function of Condition and Health (*N* = 40).

The analysis of the raw values revealed a significant effect of condition, *F*_(1,72)_ = 12.87, *p* < 0.05, ${\text{η}}_{\text{p}}^{2}$ = 0.15. Regardless of health status, participants’ motion exhibited higher magnitudes of coupling with the stimulus during the Well-flow [*M(SE)* = 0.36 (0.01)] condition than during the Sick-flow [*M(SE)* = 0.3 (0.01)] condition. Participants were influenced to a greater degree by the Well-flow stimulus than the Sick-flow stimulus. See **Figure 5** for a depiction of these results.

#### Cross-Correlation

**FIGURE 6:**
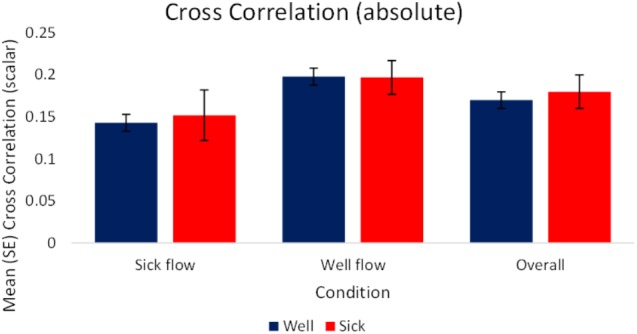
Mean (SE) Absolute cross-correlation as a function of Condition and Health (*N* = 40).

As we were interested in the magnitude of coupling rather than the type of coupling *per se*, the analysis of raw and slope values were performed on absolute values of the correlations. The analysis of the raw values revealed a significant effect of condition, *F*_(1,70)_ = 8.36, *p* < 0.05, ${\text{η}}_{\text{p}}^{2}$ = 0.11. Regardless of health status, participants’ motion exhibited higher magnitudes of coupling with the stimulus during the Well-flow [*M(SE)* = 0.198 (0.01)] condition than during the Sick-flow [*M(SE)* = 0.148 (0.01)] condition. As shown in the AMI analysis Participants were influenced to a greater degree by the Well-flow stimulus (although the correlations were in the weak range). See **Figure 6** for a depiction of these results.

#### Cross Fuzzy Entropy and Coherence

Analysis of these measures failed to reveal any significant effects.

## Discussion

In this study, we exposed participants to naturally generated optical flow produced from OPP motion. Importantly, the motion profiles represented a person who reported motion sickness and a person who remained healthy; more generally, these stimuli represented the divergent motion patterns observed in healthy and motion sick participants (Smart et al., 2014). We observed that participants’ sway patterns were differentially influenced by the two flow types and importantly different rates of reported motion sickness occurred with twice as many participants becoming motion sick in the Well-flow condition (however, the difference in rates while noteworthy, was not significant).

Similar to the findings of Smart et al. (2014), increases in the magnitude of motion (PL, EA slope) were observed in *Motion Sick* participants. However, a key difference from the previous study was revealed by examining sway PL, in that the pattern of increase was specific to the Well-flow condition. In the Sick-flow condition, we observed that *Well* participants exhibited higher magnitudes of motion (PL). In this case the increased motion may have been adaptive as the frequency of motion sickness was less than we observed in the Well-flow condition. Overall, we observed a similar divergence in EA slope (rate as with the magnitude of the participants’ motion changes) that was reported by Smart et al. (2014); with the *Motion Sick* participants showing a more rapid rise in magnitude, while the *Well* participants were slower to change and tended to have lower magnitudes of sway. It is also important to note again that the major difference between the current study and that Smart et al. (2014) is that in the previous study the open loop (playback) condition presented participants with their own motion rather than another person’s movements.

It may seem surprising that optical flow from a non-sick participant would seemingly produce more motion sickness in current participants, however, the incident rate (40%) is consistent with what was observed in response to baseline recordings (prior to any sickness) of participants’ own motion (playback condition of Smart et al., 2014) and in fact was statistically equivalent to the rate (20%) observed in the Sick-flow condition. In general the incidences of motion sickness in these conditions are consistent previous research utilizing open loop presentations of optic flow (Stoffregen and Smart, 1998; Villard et al., 2008). The data also lend support to the wave interference hypothesis posited by Stoffregen and Smart (1998) and Smart et al. (2002). This hypothesis states that like in other physical systems, when two waveforms interact, the closer in nature (amplitude, frequency) the two waveforms are the more catastrophic the interaction will be. Additionally, the differential postural response to the two types of optic flow, suggest that participants were sensitive to structural differences in the flow.

Functionally what this may indicate is that the Well-flow condition presented participants with structure that they perceived as “useful” or “usable” (i.e., sufficient to guide behavior) as evidenced by the stronger coupling (increased synchrony: AMI, CC) to the Well-flow stimulus (and to slightly higher extent for *Well* participants). This suggests that what may be occurring is that the participants are attempting to dynamically synchronize with the stimuli (evidenced by the stronger coupling exhibited in the Well-flow condition) but at times failing to do so appropriately, hence the increase in reports of motion sickness. Despite the perceived “usability” of the Well-flow, the open-loop nature of the stimulus prevents a true perception-action coupling and renders it disruptive rather than facilitative. This is supported in part by the significantly higher post immersion reports of oculomotor discomfort in the Well-flow condition. In the case of the decreased coupling observed in the Sick-flow condition, this may represent participants’ ability to discriminate abnormal or non-usable structure and their attempts to adjust their sway to compensate for the lack of “appropriate” structure. The analysis suggests that this may be the case as we observed some increases in PL, SEn, and PL_N_ for *Well* participants (although not significant).

The divergent patterns of sway characteristics between *Well* and Motion Sick participants observed in this study not only lend support to the assertion that postural motion can be used as a reliable means to assess potential motion sickness, but also supports the idea that behavior requires perceivable causal mechanisms to enact (successful) actions in support of an intended goal. The Well-flow condition seemingly provides information that participants are not only able to detect, but specify how to support an ongoing action (stable posture). It would appear, however, that providing information without any means of actualizing their function can lead to clear disruptions in behavior.

These findings also have design implications for virtual technologies as there is a resurgence in attempts to make head-mounted, first-person displays commercially viable. Motion Sickness continues to be a significant issue with the technologies that cannot be alleviated with general design improvements alone. Instead, the solutions sought should examine how one can support the emergence of “natural” perception-action relations in these virtual contexts. Doing so requires the examination of both what information/structure is available to the person as well as what actions are supported. If you are going to provide information that suggests that a given behavior or regulatory strategy is possible, the system needs to allow for that behavior/strategy to be implemented. This is important as the data from this study reveal that “open loop” presentations of information that are perceived as consequential can lead to disruptions in behavior and ill-effects. For example in many first-person perspective games, “bob and sway” are often coded into the stimulus to represent body movement. The addition of this non-controllable sway information is analogous to our experimental manipulation, and has been indicated as a factor in the emergence of motion sickness (Dong et al., 2011; Sharp, 2013). In this study, some participants were unable to modulate their behavior successfully at least in part due to the absence of consequential feedback which is characteristic of open-loop presentations. The disruptions observed in these open-looped systems illustrate the consequences of natural perception-action suppression commonly seen in VE and simulations, especially when potentially exploitable information can be acquired, but not fully utilized by the user. In short, the mere presentation of sway-like optical flow may not be sufficient for successful regulation of behavior in virtual environments, particularly without the ability to engage in real–time interaction with this optical information.

## Author Contributions

This project was a graduate project of HC and JH. LS supervised the study. HC, JH, and LS designed, conducted, and analyzed the project. Each author contributed significantly to the writing of the manuscript.

## Conflict of Interest Statement

The authors declare that the research was conducted in the absence of any commercial or financial relationships that could be construed as a potential conflict of interest.
